# 亲水相互作用色谱-静电场轨道阱高分辨质谱法测定CO_2_吸收液中9种有机胺类化合物

**DOI:** 10.3724/SP.J.1123.2022.12014

**Published:** 2023-09-08

**Authors:** Zekun TANG, Huihui WAN, Hong LI, Shaoyun CHEN, Jinfeng ZHAO, Yuming SUN, Rui CAI, Qiang XU, Hua ZHANG

**Affiliations:** 1.大连理工大学分析测试中心, 辽宁 大连 116024; 1. Instrumental Analysis Center, Dalian University of Technology, Dalian 116024, China; 2.大连理工大学化工学院, 辽宁 大连 116024; 2. School of Chemical Engineering, Dalian University of Technology, Dalian 116024, China

**Keywords:** 亲水相互作用色谱, 静电场轨道阱高分辨质谱, 有机胺类化合物, CO_2_吸收剂, hydrophilic interaction liquid chromatography (HILIC), electrostatic field orbitrap high resolution mass spectrometry, organic amine compounds, carbon dioxide (CO_2_) absorbent

## Abstract

二氧化碳(CO_2_)吸收捕集是实现我国碳达峰和碳中和目标的有效措施。有机胺类化合物被广泛用作工业回收CO_2_的吸收剂,建立有机胺类化合物的分析检测方法对于碳捕集与封存(CCS)技术和碳捕获、利用与封存(CCUS)技术的发展具有重要意义。本研究建立了以亲水相互作用色谱-静电场轨道阱高分辨质谱法同时测定CO_2_吸收液中9种有机胺类化合物的分析方法。样品以水作为溶剂,稀释后经0.22 μm尼龙滤膜过滤后,进样分析。采用Accucore HILIC色谱柱(100 mm×2.1 mm, 2.6 μm),在30 ℃条件下进行分离,流动相A为90%乙腈水溶液(含5 mmol/L甲酸铵和0.1%甲酸),流动相B为10%乙腈水溶液(含5 mmol/L甲酸铵和0.1%甲酸),梯度洗脱。采用电喷雾离子源(ESI),在正离子模式下进行测定,通过标准加入法进行定量分析。实验对比了不同色谱柱对有机胺类化合物的保留能力以及不同流动相的影响,并对方法进行了方法学验证。结果表明:9种有机胺类化合物在0.04~25000 ng/mL范围内线性关系良好,线性相关系数(*R*^2^)均≥0.9910;方法的检出限(LOD)为0.0004~0.0080 ng/mL,方法的定量限(LOQ)为0.0035~0.0400 ng/mL;在1、1.5、3倍样本浓度添加水平下,方法的平均回收率为85.30%~104.26%,相对标准偏差(RSD)为0.04%~7.95%。应用建立的方法对某项目现场样品的吸收废液进行检测,9种有机胺类化合物均能被有效检测。对实际样品进行稳定性测试,于4 ℃条件下,在48 h内RSD为0.10%~6.35%。该方法灵敏、准确、快速、简便,可为有机胺类化合物的检测提供参考,并为CO_2_捕集技术的开发和工业化应用提供有力的技术支持。

## Abstract (en)

Carbon dioxide (CO_2_) absorption and capture is an effective measure to achieve the “dual carbon” goal of carbon peak and carbon neutrality in China. Organic amine compounds are widely used in the industrial separation and recovery of CO_2_. Thus, the establishment of analytical methods for organic amine compounds is of great significance for the research and development of carbon capture and storage (CCS) technology and carbon capture, utilization and storage (CCUS) technology. In this study, a method was developed for the determination of nine organic amine compounds in CO_2_ absorption liquid by hydrophilic interaction liquid chromatography (HILIC)-electrostatic field orbitrap high resolution mass spectrometry. The sample was diluted with water and filtered through a 0.22 μm nylon membrane before sampling and analysis. An Accucore HILIC column (100 mm×2.1 mm, 2.6 μm) was used for separation at 30 ℃. Gradient elution was conducted using 90% acetonitrile aqueous solution containing 5 mmol/L ammonium formate and 0.1% formic acid as mobile phase A and 10% acetonitrile aqueous solution containing 5 mmol/L ammonium formate and 0.1% formic acid as mobile phase B. Determination was performed using an electrospray ion source (ESI) in the positive ion mode. The quantitative analysis was carried out by standard addition method. The chromatographic retention performance of different chromatographic columns and the influence of different mobile phases on the separation of the organic amine compounds were compared, and the method was validated. The results showed that the linear ranges of the nine organic amine compounds were 0.04-25000 ng/mL with the linear correlation coefficients (*R*^2^) greater than 0.9910. The limits of detection (LODs) of the method were in the range of 0.0004-0.0080 ng/mL, and the limits of quantification (LOQs) of the method were in the range of 0.0035-0.0400 ng/mL. The average recoveries of the method ranged from 85.30% to 104.26% with relative standard deviations (RSDs) of 0.04%-7.95% at the spiked levels of 1, 1.5 and 3 times sample concentration. The established method was applied to detect the absorption waste liquid of a cement plant, and nine organic amine compounds could be effectively detected. The stability of the actual sample was tested, and the RSDs were 0.10%-6.35% in 48 h at 4 ℃. The method is sensitive, rapid and accurate for the determination of the nine organic amine compounds in industrial waste water. It can provide reference for the detection of organic amine compounds, and provide strong technical support for the research and industrial application of CO_2_ capture technology.

二氧化碳(CO_2_)捕集、利用(CCS)和CO_2_捕集、利用与封存(CCUS)技术是大规模快速减排温室气体和推进我国绿色低碳发展的重要手段,是实现我国碳达峰和碳中和目标的有效措施。用化学溶剂(如链烷醇胺、碳酸盐-碳酸氢盐缓冲液、氨基酸式盐等)进行CO_2_吸收是一种有效的CO_2_分离技术,被广泛用于发电、炼钢、矿石分解等低浓度气源领域。其中以有机胺为吸收剂的CO_2_化学吸收法,因其具有吸收效率高、处理能力大的特点,被广泛应用于天然气、炼厂气、合成气及烟气等各种气体净化和CO_2_吸收工艺中^[1⇓-3]^。烟气中与CO_2_ 共存的氧气 (O_2_ )、硫氧化物 (SO*_x_* )、氮氧化物 (NO*_x_* ) 都会使有机胺发生不同机制的降解,导致溶剂的CO_2_捕集效率下降。同时,吸收剂的解吸在高温条件下进行,也会造成有机胺溶液的热降解。建立多种有机胺类化合物的分析检测方法可有效监测有机胺类CO_2_吸收剂研究与生产过程中有机胺成分及浓度水平,对于有机胺类CO_2_吸收剂碳捕集技术的研究和CCS与CCUS技术的发展具有重要的意义。

以醇胺为代表的有机胺类化合物的烷烃骨架上具有羟基和氨基,因此在水、丙酮或甲醇等极性溶剂中具有碱性且可以完全溶解。由于其独特的结构和化学特性,有机胺类化合物不仅被广泛应用于天然气、炼厂气、合成气及烟气等各种气体净化工艺中,而且被广泛用作制造表面活性剂、纺织物、除草剂和金属切削液的原料^[4,5]^。此外,醇胺化合物中以*N*-乙基二乙醇胺、*N*-甲基二乙醇胺、二乙醇胺为代表的乙醇胺化合物是《化学武器公约》禁止的氮芥的水解产物^[6]^。因此,有机胺类化合物分离分析方法的建立引起了人们广泛的关注。

文献中报道的有机胺类化合物的分析技术主要包括离子色谱^[5,7⇓⇓-10]^、液相色谱^[11]^、电泳^[12]^、基质辅助激光解吸-飞行时间质谱^[13]^、气相色谱-质谱联用^[14⇓-16]^以及液相色谱-质谱联用^[6]^。离子色谱技术主要是利用胺类化合物的阳离子离子交换性质,结合电导检测器和离子交换电雾式检测器,用于化工废水^[5]^、食品接触材料^[7]^、化妆品^[8]^和CO_2_吸收液^[9,10]^中有机胺类化合物的检测。考虑到有机胺类化合物通常无特征紫外吸收,紫外末端吸收也很弱,高效液相色谱用于这类化合物分析时,不适合选择紫外检测器。文献^[11]^利用高效液相色谱联用蒸发光散射检测器测定了乳膏中三乙醇胺的含量。此外,电泳方法被报道用于测定化妆品中三乙醇胺的含量^[12]^,基质辅助激光解吸-飞行时间质谱方法被用于氮芥水解产物的快速筛选^[13]^。色谱-质谱联用技术由于检测准确度和灵敏度高的优势被广泛用于有机胺类化合物的检测分析。气相色谱-质谱技术被报道用于尿液和血清等生物样品体系中氮芥水解有机胺类化合物的检测^[14⇓-16]^。在含水的样品体系中有机胺类化合物不易挥发、难提取,所以样品在进入气相色谱-质谱仪器分析前,需要预先进行衍生化处理^[17]^。由于有机胺类化合物在电喷雾和大气压化学电离源条件下容易电离,液相色谱-三重四极杆质谱和四极杆/飞行时间质谱技术被广泛用于有机胺类化合物的检测分析。反相色谱分离模式下这类化合物在色谱柱上的保留弱^[18,19]^,为了提高色谱保留性能,可以通过衍生化预处理方法增强分析物的疏水性^[20]^。利用有机胺类化合物的极性和电荷性质,采用亲水色谱模式分离样品,分析物在亲水相互作用色谱柱上可以获得很好的保留和分离,且省去了衍生化处理的繁琐步骤,样品前处理方便,因此亲水相互作用色谱分离模式的液相色谱-质谱方法被广泛应用于尿液和血清生物样品体系中氮芥水解醇胺产物检测方法的建立^[4,6,21⇓⇓-24]^。同时,亲水色谱模式的液相色谱-质谱技术也被应用于塑料制品包装材料^[25]^、工业废水体系中有机胺类化合物的检测分析^[4]^。

近年来,静电场轨道阱高分辨质谱技术凭借其高分辨率、高灵敏度、高通量、高扫描速率等优势,在蛋白组学、代谢组学、食品、药品、环境等方面获得了广泛的应用^[26⇓-28]^。本研究建立了亲水相互作用色谱-静电场轨道阱高分辨质谱法测定CO_2_吸收液中9种有机胺类化合物的分析方法,并进行了详细的方法学验证。

## 1 实验部分

### 1.1 仪器,试剂与材料

Q Exactive plus超高效液相色谱-静电场轨道阱质谱联用系统(美国Thermo Scientific公司),包括Vanquish超高效液相色谱系统和Orbitrap高分辨质谱仪,Xcalibur 4.4软件用于仪器控制和数据处理;Milli-Q超纯水系统(美国Billercia公司);尼龙滤膜(0.22 μm,天津博纳艾杰尔科技有限公司); ME 204型电子天平(瑞士Mettler Toledo公司); Accucore HILIC色谱柱(100 mm×2.1 mm, 2.6 μm)、Hypersil GOLD C18色谱柱(100 mm×2.1 mm, 3 μm)(美国Thermo Scientific公司)。

甲醇、乙腈(色谱纯,美国Thermo Scientific公司),甲酸(色谱纯,北京迪科马科技有限公司),甲酸铵(色谱级,德国Honeywell公司),乙醇(分析纯,天津市富宇精细化工有限公司)。

标准品(纯度>99%): 2-氨基-2-甲基-1,3-丙二醇(2-amino-2-methyl-1,3-propanediol, AMPD)、哌嗪(piperazine, PZ)、*N*,*N*-二甲基乙醇胺(*N*,*N*-dimethylethanolamine, DMEA)、*N*-乙基乙醇胺(*N*-ethyl ethanolamine, EMEA)、羟乙基乙二胺(hydroxyethyl ethylenediamine, AEEA)、*N*,*N*-二甲基环己胺(*N*,*N*-dimethylcyclohexylamine, DMCHA)、*N*-甲基单乙醇胺(*N*-methyl monoethanolamine, MMEA)、*N*-甲基二乙醇胺(*N*-methyldiethanolamine, MDEA)、二乙氨基乙醇(diethylaminoethanol, DEAE)均购自上海阿拉丁生化科技股份有限公司。9种有机胺类化合物的化学结构信息如图1所示。

**图 1:**
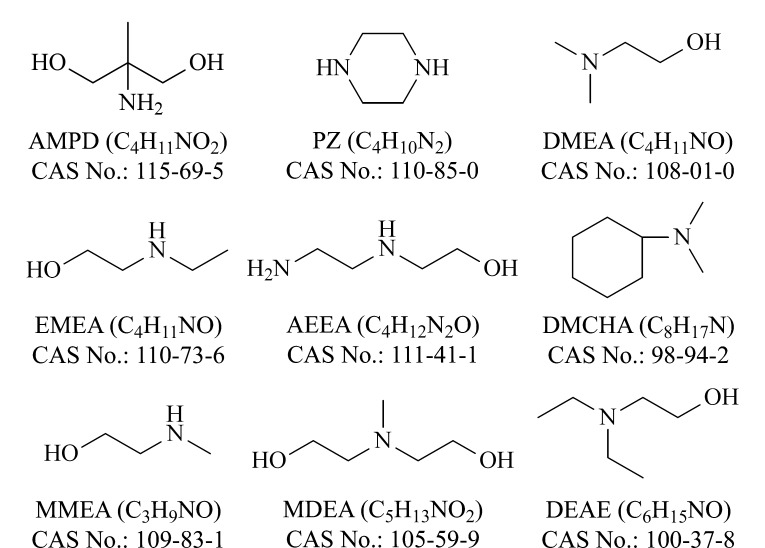
9种有机胺类化合物的化学结构信息

### 1.2 溶液的配制

分别准确称取适量9种有机胺类化合物,用乙醇配制成质量浓度为500 μg/mL的标准储备液,保存于4 ℃冰箱中备用。

分别准确量取适量标准储备液,配制成质量浓度分别为25000、5000、1000、200、40、4、1、0.20和0.04 ng/mL的系列混合标准工作液。

### 1.3 样品前处理

本实验所用的3份CO_2_吸收液来自工厂的某项目现场样品。取50 μL工厂中的废液于10 mL容量瓶中定容,再取100 μL稀释后的溶液于10 mL容量瓶中,将废液稀释成原浓度的1/20000,经过0.22 μm滤膜过滤后,进行液相色谱-质谱分析。

### 1.4 仪器分析条件

#### 1.4.1 色谱条件

色谱柱选用Accucore HILIC(100 mm×2.1 mm, 2.6 μm);柱温:30 ℃;流动相A为90%乙腈水溶液(含5 mmol/L甲酸铵和0.1%甲酸),流动相B为10%乙腈水溶液(含5 mmol/L甲酸铵和0.1%甲酸)。梯度洗脱程序:0~7 min, 100%A~80%A; 7~11 min, 80%A~50%A; 11~12 min, 50%A~100%A; 12~22 min, 100%A。流速:0.2 mL/min;进样量:5 μL。

#### 1.4.2 质谱条件

采用电喷雾电离(ESI)源,正离子模式,喷雾电压3.6 kV,归一化碰撞能量(NCE)20、40和60,离子传输管温度320 ℃;辅助气温度100 ℃;鞘气35 arb,辅助气10 arb;检测方式为全扫描/数据依赖二级扫描(Full-MS/dd-MS^2^)模式;Full-MS分辨率70000,自动增益控制(AGC target)3×10^6^,最大离子注入时间(Maximum IT)50 ms, Full-MS扫描范围*m/z* 60~600; dd-MS^2^分辨率17500, AGC target 2×10^6^, Maximum IT 100 ms,隔离窗口(Isolation window)2.0。9种有机胺类化合物的质谱信息见表1,以分析物的母离子为定量离子,以产物离子为定性离子。

**表 1:** 9种有机胺类化合物的质谱信息

No.	Compound	t_R_/min	Ion mode	Predicated ion (m/z)	Observed ion (m/z)	Product ions (m/z)
1	AMPD	5.38	[M+H]^+^	106.08626	106.08691	88.07599, 71.04965
2	PZ	10.40	[M+H]^+^	87.09167	87.09198	85.07635, 70.07570
3	DMEA	5.78	[M+H]^+^	90.09134	90.09156	72.08132, 70.06561
4	EMEA	6.78	[M+H]^+^	90.09134	90.09156	72.08130, 70.06569
5	AEEA	10.08	[M+H]^+^	105.10224	105.10227	88.07602, 70.06571
6	DMCHA	5.13	[M+H]^+^	128.14338	128.14301	83.08586, 55.05496
7	MMEA	6.18	[M+H]^+^	76.07570	76.07606	58.06579, 56.05017
8	MDEA	6.58	[M+H]^+^	120.10191	120.10165	102.09143, 58.06580
9	DEAE	6.10	[M+H]^+^	118.12264	118.12241	100.11222, 72.08123

## 2 结果和讨论

### 2.1 色谱条件优化

#### 2.1.1 色谱柱的选择

本实验对比了Hypersil GOLD C18(100 mm×2.1 mm, 3 μm)和Accucore HILIC(100 mm×2.1 mm, 2.6 μm)两种色谱柱。实验结果表明:采用Hypersil GOLD C18色谱柱(100 mm×2.1 mm, 1.9 μm)时,9种目标化合物因极性较强,在反相色谱模式下保留很弱,不能很好地分离。选用Accucore HILIC色谱柱(100 mm×2.1 mm, 2.6 μm)时, 9种目标化合物在亲水模式下得到了较好的保留和分离。因此,本实验选择在Accucore HILIC色谱柱(100 mm×2.1 mm, 2.6 μm)上进行色谱分离分析。

#### 2.1.2 流动相的选择

在分析过程中,流动相中有机酸的添加会影响目标化合物的保留时间、色谱峰形等。本实验比较了有机相为90%乙腈水溶液(含5 mmol/L甲酸铵)、水相为10%乙腈水溶液(含5 mmol/L甲酸铵)和有机相、水相均添加0.1%甲酸时分析物的色谱保留。

实验结果表明,当流动相不添加0.1%甲酸时,PZ与AEEA化合物的色谱峰拖尾严重,且保留时间太长;当添加0.1%甲酸时,所有待测组分的色谱峰具有良好的峰形,PZ与AEEA的保留时间分别为10.40和10.08 min。因此,选择有机相90%乙腈水溶液(含5 mmol/L甲酸铵和0.1%甲酸)、水相10%乙腈水溶液(含5 mmol/L甲酸铵和0.1%甲酸)作为流动相,以梯度模式进行洗脱。9种有机胺类化合物的提取离子流色谱图见图2a。

**图 2:**
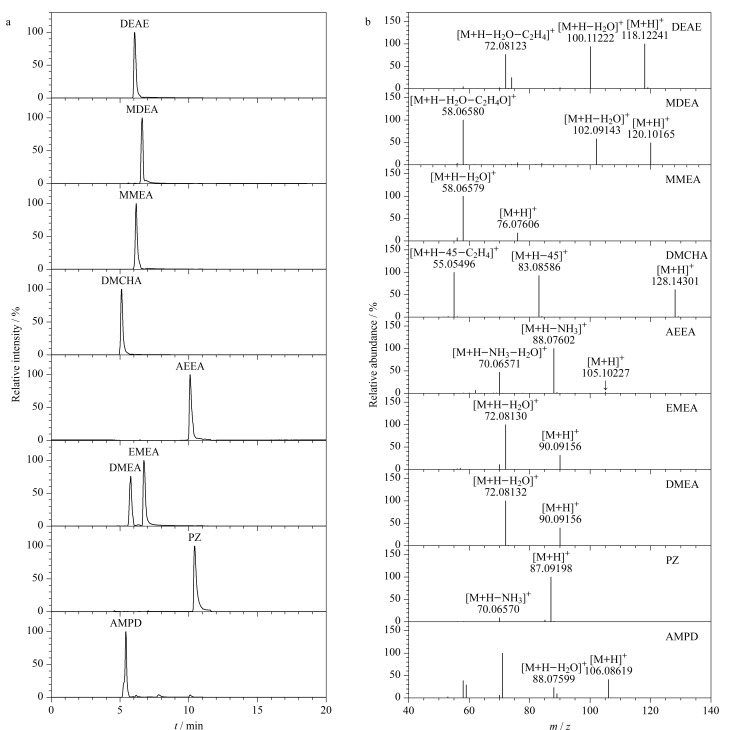
9种有机胺类化合物的(a)提取离子流色谱图和(b)二级质谱图

### 2.2 质谱参数优化

本实验使用超高效液相色谱-静电场轨道阱高分辨质谱进行目标化合物的检测,首先采取单针进样的方式,对9种目标化合物的混合标准工作液(5.0 μg/mL)进行质谱条件的优化。在目标化合物进入质谱后,通过一级全扫描,得到目标化合物的母离子,所有目标化合物均可以产生稳定的[M+H]^+^离子,因此,对所有的目标化合物采取正离子电喷雾模式进行一级全扫描。确定出目标化合物的母离子后,通过优化气流、温度、电压等参数,进行质谱条件的优化。优化后的质谱条件如1.4.2节所示,采用Full-MS/dd-MS^2^模式,9种有机胺类化合物的高分辨二级质谱图、母离子和碎片离子信息^[22]^如图2b所示。

### 2.3 线性范围、检出限和定量限

基质效应指的是目标化合物的离子化效率会受到样品体系中基质的干扰,造成质谱信号被增强或抑制,从而影响质谱定量分析的准确性。为了减少基质效应,实验采取标准加入法建立标准曲线,取200 μL稀释后的工厂CO_2_吸收液,向其中加入等量的混合标准工作液,配制0.04~25000 ng/mL的基质匹配标准溶液。在已优化的条件下,取基质匹配标准溶液进行分析,以9种有机胺类化合物的质量浓度为横坐标*x*,以目标分析物母离子的色谱峰面积为纵坐标*y*,绘制标准曲线。如表2结果所示,9种目标化合物在一定范围内线性关系良好,相关系数(*R*^2^)≥0.9910。以信噪比(*S/N*)≥3的质量浓度为检出限(LOD),*S/N*≥10的质量浓度为定量限(LOQ),逐级稀释标准溶液,得到9种化合物的LOD和LOQ。实验结果表明:方法的检出限范围为0.0004~0.0080 ng/mL,方法的定量限范围为0.0035~0.0400 ng/mL。

**表 2:** 9种有机胺化合物的线性方程、相关系数、检出限和定量限

No.	Compound	Linear range/ (ng/mL)	Linear equations				R^2^			LOD/ (ng/mL)		LOQ/ (ng/mL)
			Sample 1	Sample 2	Sample 3		Sample 1	Sample 2	Sample 3			
1	AMPD	0.04-25000	y=1.3005x+805166	y=1.6144x+17965	y=1.6472x+116565		0.9984	0.9910	0.9926		0.0030	0.0250
2	PZ	0.04-25000	y=3.3012x+5.25675	y=3.4960x+5.03065	y=4.1866x+5.18668		0.9995	0.9987	0.9981		0.0030	0.0250
3	DMEA	0.04-25000	y=2.8453x+3.36330	y=4.0884x+6.60093	y=4.1026x+6.56735		0.9989	0.9980	0.9981		0.0030	0.0350
4	EMEA	0.04-25000	y=2.9006x+6.90375	y=3.77495x+4.92543	y=4.0225x+4.99919		0.9943	0.9979	0.9953		0.0020	0.0100
5	AEEA	0.04-25000	y=122330x+3.07125	y=123380x+3.1961	y=104089x+7.0628		0.9983	0.9986	0.9993		0.0004	0.0035
6	DMCHA	0.04-25000	y=4.1194x+1.73009	y=4.75736x+1.35725	y=5.41105x+1.32278		0.9999	0.9974	0.9972		0.0020	0.0100
7	MMEA	0.04-25000	y=1.1483x+5.12991	y=1.97590x+2.34735	y=1.98902x+2.01331		0.9996	0.9932	0.9926		0.0080	0.0400
8	MDEA	0.04-25000	y=556888x+3.21387	y=565067x+3.35909	y=597026x+1.14919		0.9991	0.9987	0.9957		0.0004	0.0035
9	DEAE	0.04-25000	y=4.0549x+1.22349	y=5.68936x+1.11871	y=5.49765x+2.46986		0.9910	0.9947	0.9994		0.0050	0.0200

y: peak area; x: mass concentration, ng/mL.

### 2.4 回收率与精密度

向样本中分别加入样本水平、1.5倍样本水平、3倍样本水平的基质匹配标准溶液,每个水平进行5次平行试验,结果见表3。9种有机胺类化合物的回收率为85.30%~104.26%, RSD为0.04%~7.95%,能够满足实际样品的检测需求。

**表 3:** 9种有机胺类化合物在不同基质中的添加回收率和相对标准偏差(n=5)

Compound		Sample 1								Sample 2								Sample 3					
		Added/(ng/mL)		Recovery/%		RSD/%				Added/(ng/mL)		Recovery/%		RSD/%				Added/(ng/mL)		Recovery/%		RSD/%	
AMPD		0.60		89.24		0.23				0.20		97.75		1.58				0.30		89.84		5.60	
		0.90		90.94		0.07				0.30		103.55		1.21				0.45		91.20		1.51	
		1.80		95.17		0.16				0.60		95.61		0.19				0.90		91.75		1.06	
PZ		6.00		97.23		0.16				5.00		90.74		5.16				30		91.13		1.21	
		9.00		90.59		0.51				7.50		91.65		3.59				45		92.58		0.57	
		18.0		96.57		1.10				15.0		92.99		1.71				90		95.36		1.30	
DMEA		2.00		95.31		1.31				5.00		98.29		0.2				4.00		92.03		3.22	
		3.00		92.84		1.63				7.50		100.91		0.3				6.00		97.60		0.13	
		6.00		93.20		0.22				15		97.38		0.6				12.0		98.85		1.46	
EMEA		1.00		104.26		0.14				0.20		91.25		1.50				5.00		97.68		0.73	
		1.50		96.26		0.21				0.30		96.74		0.37				7.50		96.72		0.18	
		3.00		92.34		0.31				0.60		91.59		0.11				15.0		97.32		0.04	
AEEA		1400		99.68		2.17				6.00		97.56		2.65				300		97.78		1.25	
		2100		97.88		1.76				9.00		94.91		3.98				1000		98.98		0.20	
		4200		97.73		1.37				18.0		95.37		7.95				1500		95.96		0.83	
DMCHA		0.70		85.30		3.50				0.50		87.30		3.72				0.20		87.60		5.43	
		1.05		92.16		3.20				0.75		96.02		5.58				0.30		92.59		3.28	
		2.10		90.34		0.12				1.50		103.03		1.16				0.60		95.20		1.66	
MMEA		100		98.74		4.19				5.00		98.65		2.09				6.00		90.92		1.95	
		150		99.64		0.69				7.50		95.62		2.33				9.00		92.61		0.74	
		300		97.48		2.33				15.0		90.25		1.63				18.00		92.47		1.06	
MDEA		500		98.62		3.20				350		97.58		2.16				250		96.01		2.78	
		750		97.79		0.54				525		92.30		1.15				375		96.80		0.95	
		1500		94.69		1.13				1750		92.09		3.06				1250		91.93		1.10	
DEAE		3.00		93.69		4.02				0.50		102.96		2.35				15		93.74		3.38	
		4.50		95.48		0.81				0.75		98.10		3.53				20		95.95		1.15	
		9.00		95.05		1.11				1.50		94.17		7.05				50		91.98		0.17	

### 2.5 实际样品检测

用本方法对3份工厂某项目现场的CO_2_吸收废液进行检测,结果如表4显示,只有1种CO_2_吸收废液中未检测到EMEA,其余的有机胺化合物在吸收废液均能检测到。为了研究有机胺的稳定性,考察了3份实际样本在4 ℃下分别放置0、12、24、36、48 h条件下,9种不同有机胺目标分析物的含量。结果见表4,实验数据表明,在48 h内,在4 ℃下,9种有机胺类化合物在不同基质中的RSD范围为0.10%~6.35%,稳定性良好。

**表 4:** 实际样品中9种有机胺类化合物的稳定性

Compound	Matrix	Contents/(ng/mL)					RSD/%
		0 h	12 h	24 h	36 h	48 h	
AMPD	sample 1	0.584	0.574	0.569	0.573	0.577	0.97
	sample 2	0.163	0.164	0.166	0.171	0.182	4.60
	sample 3	0.323	0.321	0.338	0.309	0.311	3.61
PZ	sample 1	6.170	6.351	5.872	6.144	6.208	2.83
	sample 2	4.522	4.514	4.494	4.601	4.504	0.94
	sample 3	26.944	26.816	25.872	26.144	25.988	1.87
DMEA	sample 1	2.605	2.602	2.487	2.727	2.631	3.28
	sample 2	0.229	0.248	0.239	0.249	0.250	3.69
	sample 3	3.616	3.633	3.487	3.727	3.599	2.38
EMEA	sample 1	1.000	0.904	0.989	1.006	1.082	6.35
	sample 2	0.178	0.179	0.188	0.183	0.185	2.28
	sample 3	0					
AEEA	sample 1	1374.88	1374.25	1373.55	1375.83	1377.25	0.10
	sample 2	6.275	6.351	6.475	5.998	6.051	3.24
	sample 3	318.875	317.153	316.475	322.998	319.817	0.81
DMCHA	sample 1	0.696	0.754	0.666	0.669	0.709	5.12
	sample 2	0.525	0.537	0.544	0.544	0.518	2.19
	sample 3	0.182	0.177	0.190	0.185	0.187	2.70
MMEA	sample 1	113.395	114.752	113.397	112.037	115.021	1.06
	sample 2	4.514	4.539	4.382	4.622	4.489	1.93
	sample 3	6.064	6.189	6.382	5.962	6.003	2.77
MDEA	sample 1	453.847	452.270	456.942	453.330	455.201	0.40
	sample 2	336.094	335.858	340.094	332.330	334.236	0.86
	sample 3	246.459	243.952	238.094	257.330	247.454	2.83
DEAE	sample 1	2.593	2.592	2.627	2.603	2.612	0.56
	sample 2	0.472	0.481	0.518	0.468	0.478	4.14
	sample 3	13.480	13.478	13.318	13.468	13.502	0.55

## 3 结论

本研究建立了基于亲水相互作用色谱-静电场轨道阱高分辨质谱法同时测定CO_2_吸收液中9种典型有机胺类化合物的分析方法。该方法定量准确,重复性好,操作简便,可应用于天然气、炼厂气、合成气及烟气等各种气体净化和CO_2_吸收工艺中,能够有效监测有机胺类CO_2_吸收剂研究与生产过程中的有机胺成分及浓度水平,可为有机胺类CO_2_吸收剂碳捕集技术的开发和工业化应用提供分析检测技术支持。
